# Prevention and Management of the Post-Thrombotic Syndrome

**DOI:** 10.3390/jcm9040923

**Published:** 2020-03-27

**Authors:** Ilia Makedonov, Susan R. Kahn, Jean-Philippe Galanaud

**Affiliations:** 1 Division of Internal Medicine, Department of Medicine, University of Ottawa, Ottawa, ON K1H 8L6, Canada; 2 Center for Clinical Epidemiology, Jewish General Hospital/Lady Davis Institute; Division of Internal Medicine, Department of Medicine, McGill University, Montreal, QC H3A 1A1, Canada; susan.kahn@mcgill.ca; 3 Department of Medicine, Sunnybrook Health Sciences Centre and University of Toronto, Toronto, ON M4N 3M5, Canada; Jean-Philippe.Galanaud@sunnybrook.ca

**Keywords:** deep vein thrombosis, post-thrombotic syndrome, elastic compression stockings, catheter-directed thrombolysis, low-molecular-weight heparins

## Abstract

The post-thrombotic syndrome (PTS) is a form of chronic venous insufficiency secondary to prior deep vein thrombosis (DVT). It affects up to 50% of patients after proximal DVT. There is no effective treatment of established PTS and its management lies in its prevention after DVT. Optimal anticoagulation is key for PTS prevention. Among anticoagulants, low-molecular-weight heparins have anti-inflammatory properties, and have a particularly attractive profile. Elastic compression stockings (ECS) may be helpful for treating acute DVT symptoms but their benefits for PTS prevention are debated. Catheter-directed techniques reduce acute DVT symptoms and might reduce the risk of moderate–severe PTS in the long term in patients with ilio-femoral DVT at low risk of bleeding. Statins may decrease the risk of PTS, but current evidence is lacking. Treatment of PTS is based on the use of ECS and lifestyle measures such as leg elevation, weight loss and exercise. Venoactive medications may be helpful and research is ongoing. Interventional techniques to treat PTS should be reserved for highly selected patients with chronic iliac obstruction or greater saphenous vein reflux, but have not yet been assessed by robust clinical trials.

## 1. Introduction

The post-thrombotic syndrome (PTS) is a form of chronic venous insufficiency (CVI) that occurs secondary to a prior deep vein thrombosis (DVT) [1]. It develops in 20–50% of patients after a proximal DVT [2]. Like any form of CVI, PTS is attributed to ambulatory venous hypertension and this can be caused either by residual venous obstruction, valvular damage, or both. The typical symptoms are leg heaviness, pain, edema and pruritus, which tend to be worse by end of day. Trophic changes range from hyperpigmentation to venous ulceration, in the most severe form [3]. PTS is not a lethal condition but it negatively impacts quality of life, with an effect comparable to that of other chronic conditions such as heart failure or diabetes mellitus [4,5,6]. This review focuses on clinically relevant PTS management questions and addresses controversies within the field.

## 2. Case One—Prevention of PTS

A 57 year old man presents to the emergency room with an unprovoked left-sided iliofemoral DVT. There are no respiratory symptoms. The clot extends from the common iliac vein down to the popliteal vein. The leg is very swollen and tender and symptoms started 3 days ago. There is diffuse erythema and hyperpigmentation in both legs. Distal arterial pulses are present. His weight is 105 kg and height is 180 cm (BMI 32). His medical history includes hypertension and a previous right-sided proximal DVT after a long-haul flight 5 years ago. This was treated with anticoagulation for 6 months. CBC, creatinine and coagulation parameters are unremarkable. He has medication coverage through work.

### 2.1. Can PTS Be Diagnosed at This Point?

Even though the acute phase of VTE can present with similar symptoms to PTS (pain, edema, erythema), it is impossible to diagnose PTS at this point as PTS refers only to chronic venous changes following DVT. The acute symptoms need to resolve and the diagnosis can only be made 3–6 months afterwards. For this patient, the focus is on estimating the risk of PTS and prevention.

### 2.2. What Is This Patient’s Risk of Developing PTS?

Three risk models have been developed for predicting PTS [7,8,9]. The models are based on known risk factors for PTS such as presence of iliac DVT, obesity, number of DVT-related symptoms and signs, and baseline CVI prior to DVT (Table 1). Scores for all models can be calculated from readily available clinical information. Two of these models have been externally validated [7,9]. All models appear to have similar performance characteristics, with areas under curve (AUC) of 0.66, 0.71 and 0.71, respectively, for the Rabinovich, Amin and Méan models respectively [7,8,9].

The patient in our vignette would be predicted as having a 25%, 40% or 80.7% risk of PTS based on the Rabinovich, Amin and Méan models, respectively. This discrepancy likely occurs because of differences in the models’ derivation datasets. The Rabinovich model is based on the SOX randomized control trial (RCT), which defined PTS based on the Ginsberg measure (predicting moderate to severe PTS more than mild PTS) [10]. This trial also excluded patients with previous DVT, an important risk factor for PTS and for severe PTS [11], which is, therefore, not incorporated in the model. The Amin model is based on a dataset from the Netherlands, and a Villalta score ≥ 5 at 6 months was used to define PTS. The Méan model is based on the SWITCO65+ cohort, and defined PTS as a Villalta score ≥ 5 within 24 months. This study excluded all patients under 65 years of age (around half of all VTE’s occur under this age), and its PTS risk estimates tend to be skewed to the higher side (likely reflecting the fact that age itself is a risk factor). Because of the variability in these estimates, care should be used when interpreting them or communicating them to patients. The models may be more useful for estimating whether a patient is high or low risk than for obtaining a specific point estimate.

The patient in our vignette scores at the high range of all three risk prediction models. Therefore, it will be important to ensure that he receives optimal care to reduce his risk of PTS.

### 2.3. What Medical Measures should Be Used for the Prevention of PTS?

#### 2.3.1. Does Choice of Anticoagulant Influence the Risk of Post-Thrombotic Syndrome?

Patients with proximal DVT or pulmonary embolism (PE) are treated with therapeutic doses of anticoagulants to prevent clot extension and embolization [12]. However, even though it is not the main purpose of treatment, anticoagulation also strongly influences the risk of developing PTS. Historical retrospective series assessing the risk of PTS before and after the development of anticoagulation to treat DVT suggest that anticoagulation reduces the risk of PTS by up to 78% [13,14]. Therefore, anticoagulant treatment itself is the best tool for reducing the risk of PTS. PTS guidelines do not recommend a specific anticoagulant after DVT for PTS prevention but data suggest that the type and quality of anticoagulant could influence the risk of PTS [15]. 

##### Vitamin K Antagonists (VKAs)

VKAs have been the standard treatment of DVT for more than half a century [16]. It has been shown that the quality of VKA treatment influences the risk of developing PTS [17]. Patients treated with warfarin who develop PTS have a 2.43 odds ratio of having had subtherapeutic international normalized ratio (INR) tests more than half of the time during the initial 3 months of treatment [18]. Therefore, it is critical that patients treated with VKA have judicious INR control during the initial months of therapy for PTS prevention purposes. 

##### Low-Molecular-Weight Heparins (LMWH)

Physiologic and clinical data suggest that LMWH could be superior to VKA for the prevention of PTS. In the Home-LITE study comparing tinzaparin to warfarin for 3 months in 480 patients with proximal DVT, the risk of developing signs and symptoms of PTS was lower in patients who had been treated with tinzaparin (OR 0.77, 95% CI 0.66–0.91) [19]. Other clinical studies found better rates of venous recanalization in patients treated with LMWH vs. VKA (risk ratio 0.66, (0.57–0.77)) [20,21,22,23,24,25,26,27]. Interestingly, this greater effectiveness of LMWH was not achieved via a lower rate of VTE recurrence [19]. One mechanism that could explain such a finding is the anti-inflammatory property of LMWH [28]. Inflammation is believed to have a central role in the development of PTS, and elevated inflammatory marker levels have been associated with an increased risk of PTS [29]. Anti-inflammatory effects of LMWH have been demonstrated in animal studies showing reduced venous wall inflammation [30], faster endothelialisation [31] and reduced fibrosis [32] after treatment with LMWH. Even though there is a good pathophysiological basis to support the beneficial effect of LMWH vs. VKA for PTS prevention, it should be kept in mind that the previously mentioned studies have limitations, including the use of patient self-reported, non-validated tools for PTS assessment [19]. Therefore, confirmatory studies are needed. 

If LMWH seems superior to VKA for PTS prevention, little is known regarding the effect of direct oral anticoagulants (DOACs), the new standard treatment of DVT [12], with respect to PTS prevention. Indeed, PTS was not an outcome of studies that validated the use of DOACs for the treatment of acute VTE [33,34,35,36]. Since the publication of these trials, there has been growing evidence to suggest that DOACs could also be superior to VKAs for PTS prevention. Several retrospective studies with sample sizes ranging from 100 to 1345 patients have found a reduced risk of PTS in patients treated with DOACs [37,38,39,40]. A large registry-based study of nearly 20,000 patients from Europe also found a trend towards decreased risk of PTS with DOAC treatment compared to VKA treatment, but this result was not statistically significant (HR 0.88, (0.66–1.17)) [41]. Importantly, this study used proxy measures for the diagnosis of PTS based on ICD diagnostic codes, which may limit the accuracy of their dataset. The long-term EINSTEIN follow-up ancillary study also showed a trend to decreased risk of PTS with rivaroxaban, but this was again not statistically significant (HR 0.76, (0.51–1.13)). Unfortunately, the groups were imbalanced, with iliofemoral DVTs, which are the DVTs at highest risk of PTS, present in 67% of patients in the VKA group but only 57% of patients in the rivaroxaban group [42]. It remains unknown whether DOACs, which do not possess glycan chains and, therefore, do not have anti-inflammatory properties, are equivalent to LMWH with respect to PTS prevention [28]. The ongoing Tinzaparin Lead-In to Prevent the Post-Thrombotic Syndrome Study (TILE) pilot trial is comparing tinzaparin to rivaroxaban in patients with acute ilio-femoral DVT (i.e., high risk of PTS), and will address this important issue.

Overall, we do not prefer any one anticoagulant over others for the prevention of PTS. However, if a patient has an extensive DVT and we predict a high risk of PTS, we usually offer a 2–3 week lead-in period of LMWH before switching to an oral agent.

#### 2.3.2. Should Duration of Anticoagulation Be Extended to Reduce the Risk of PTS?

The Extended AntiCoagulation Treatment for venous thromboembolism (ExACT) trial evaluated whether extended anticoagulation reduces the risk of PTS and increases quality of life [43]. This trial randomized 281 participants with unprovoked proximal VTE who had already completed 3 months of anticoagulation to either discontinue anticoagulation or continue for a total of 2 years. The mean adjusted Villalta scores were 5.0 and 5.09 for the control and extended anticoagulation groups, respectively (*p* = 0.9). The VEINES-QoL and EQ-5D-3L quality of life scores were also not significantly different between groups. Shulman et al. analogously showed in a 10 year follow-up study of the Duration of Anticoagulation (DURAC) trial that the risk of PTS was not significantly different between the 6 week and 6 month arms [44]. Therefore, we do not recommend extended anticoagulation for the primary purpose of prevention of PTS. Extended anticoagulation should be offered based on existing guidelines [12] and calculators of VTE recurrence risk [45]. The patient in our vignette would receive indefinite anticoagulation because he developed a recurrent, now unprovoked, VTE.

#### 2.3.3. Should Statins Be Prescribed to Reduce the Risk of PTS?

Statins have anti-inflammatory properties, and one animal study showed that statin treatment led to reduced thrombus size and venous wall scarring [46]. The mechanisms are felt to include an inhibition of adhesion molecules that recruit inflammatory cells, induction of cellular accumulation of nitric oxide synthase and an inhibition of procoagulant factors [47]. A retrospective study in humans showed that statin use was associated with a higher odds of thrombus resolution or improvement (OR 3.23, (1.32–7.87)) [48]. A randomized trial of 234 patients showed that statin use reduced the rate of PTS (38.3% and 48.5% in the control and statin groups respectively, *p* = 0.02), but that quality of life was not different between groups [49]. There is currently insufficient data to support statin use for the prevention of PTS but it may constitute an interesting option as, unlike anti-inflammatory drugs, statins are not associated with an increased risk of bleeding [50]. The ongoing SAVER RCT aims to answer the question more definitively (Clinicaltrials.gov identifier NCT02679664). This open label trial is randomizing patients with a recent VTE to standard treatment plus rosuvastatin or standard treatment alone.

### 2.4. Should Elastic Compression Stockings Be Used to Prevent Post-Thrombotic Syndrome?

The utility of ECS for prevention of PTS is controversial. Several randomized open label European trials found impressive 50% relative reductions in the risk of PTS with use of ECS for 2 years after DVT [51,52]. Other randomized trials were less relevant because they randomized patients months after the acute event [53,54]. Musani et al. reported a 52% relative risk (40–67%) of PTS with ECS in their meta-analysis from 2010 [55]. This early data served as the basis of practice guidelines for many years [56]. However, the only double-blind trial of ECS found that the risk of PTS was not reduced by ECS (HR 1.1 (0.73–1.8)) [57], although compliance in this trial was suboptimal (56% of participants wore stockings 3 or more days per week at two years). Meta-analyses have also been diverged. Several meta-analyses found that ECS may be beneficial [58,59,60], whereas others did not [61,62,63]. For a comprehensive overview of existing trials and their quality, please see the Cochrane review by Appelen et al. [58]. Regardless, all meta-analyses noted that the level of evidence was low and heterogeneity was high, suggesting clinical equipoise. In such situations, we believe that clinician judgment and patient preference should take precedence. While daily use of ECS can be burdensome to patients, adverse effects are rare, with around 2–6% of patients experiencing pruritus in the largest studies [51,57].

If a clinician does believe in the utility of long-term use of ECS to prevent PTS and the patient is motivated to attempt this, the next questions are: when should they be applied, how long should patients wear them, which type should be used, which strength is optimal, and what is the role of inelastic wrappings?

#### 2.4.1. When Should ECS Be Applied?

The SOX trial did not show any benefit of ECS for treating early symptoms, but the first follow-up occurred at 14 days (randomization occurred an average of 4.7 days after DVT diagnosis). This may have been too far along from the acute phase to show a benefit [64]. In the Cactus double-blind RCT of patients with symptomatic distal DVT, those who were prescribed ECS had better pain scores at 1 week than those who were not, suggesting a beneficial effect [65]. Even though PTS guidelines do not support the use of ECS for PTS prevention, they suggest the use of ECS for management of acute DVT symptoms [15]. Regarding prevention of PTS, a substudy of the IDEAL-DVT study showed that patients who were randomized in centers that applied ECS early, or within 24 h of diagnosis, had a lower risk of residual venous obstruction [66], and those with reduced residual venous obstruction had a lower risk of PTS. However, groups were not well balanced, with more femoral-popliteal than common femoral DVTs in the early compression group. Overall, immediate application of ECS has some support for the management of acute DVT symptoms, and potentially for preventing PTS. 

#### 2.4.2. For How Long Should ECS Be Used after a DVT?

Two years of treatment has been standard practice since the historical study by Brandjes et al. [52]. A shorter duration was first investigated by Mol et al. in the OCTAVIA study [67]. In this open label RCT, 518 patients who were adherent to ECS and free from PTS one year after a proximal DVT were randomized to stop ECS or continue ECS for an additional year. It was found that stopping ECS was not non-inferior to continuing ECS (19.9% and 13.0% risk of PTS in the stop-ECS and continue-ECS groups; absolute difference of 6.9% with a 95% CI upper limit of 12.3% exceeding the prespecified 10%). The IDEAL-DVT study addressed the same issue but with a different design: to be eligible to stop ECS before 2 years (i.e., as early as the 6th month post-DVT), patients were required to have 2 consecutive Villalta scores < 5 [68]. With this protocol, the rate of PTS was similar between groups (29% and 28% in the individualized and standard duration groups), and 62% of patients were able to safely stop ECS before one year without requiring reinstatement. Thus, it should be safe to stop ECS before 2 years in a large subgroup of DVT patients, provided that they have a period of symptom stability before stopping.

#### 2.4.3. Are below Knee ECS Equivalent to above Knee ECS?

Another important question regarding ECS use is stocking length. In the Canano study, Prandoni et al. randomized 367 patients with a first proximal DVT to receive either 30–40 mmHg above-knee or below-knee ECS for 36 months [69]. It was demonstrated that both types of stockings were equivalent in terms of PTS prevention (overall PTS: 35.6% vs. 32.6%, severe PTS: 2.3% vs. 2.2% in the below- and above-knee groups, respectively). However, below-knee ECS were better tolerated with fewer side effects (27.3% vs. 40.7% rates of adverse events in the below- and above-knee groups, *p* = 0.02), resulting in better compliance (82.6% vs. 66.7% in the below- and above-knee groups, *p* = 0.003). Therefore, we recommend below-knee ECS to those patients wishing to use ECS.

Overall, the two remaining questions regarding ECS for PTS prevention are whether or not ECS are truly effective for preventing PTS, which can only be answered via another double-blind trial; and whether lower compression stockings could be superior to higher compression ones via better compliance. The latter question is the objective of the ongoing double-blinded CELEST study that compares 20–30 mmHg vs. 30–40 mmHg ECS for 2 years in patients with proximal DVT (ClinicalTrials.gov identifier NCT01578122).

This patient would be prescribed ECS because of his acute DVT symptoms but also because he has symptoms and signs of pre-existing CVI (hyperpigmentation).

#### 2.4.4. What Is the Role of Inelastic Wrappings?

Inelastic wraps are sometimes used in the acute phase of DVT until leg circumference decreases, at which point patients are transitioned to ECS. There is some hemodynamic evidence that inelastic wraps achieve better venous return than ECS, possibly through a larger increase in venous pressure with standing than with ECS [70]. However, this theoretical advantage has not been linked to a reduced incidence of PTS, and we do not preferentially recommend inelastic wraps over ECS. A clinical trial is ongoing to clarify the role of inelastic wraps in reducing the rate of PTS, but it does not have a comparator arm (ClinicalTrials.gov identifier NCT03368313).

### 2.5. Should Early Thrombus Removal Techniques Be Used to Reduce the Risk of PTS?

Early thrombus removal techniques include systemic thrombolysis, catheter-directed thrombolysis (CDT) and pharmacomechanical catheter-directed thrombolysis (PCDT) [71]. Systemic thrombolysis has been associated with an unacceptable increased risk of bleeding and particularly of intracranial haemorrhage, and is, therefore, seldom used [72,73,74].

Early studies of locoregional techniques have either been retrospective [75], non-randomized [76] or did not evaluate clinically relevant end points [77]. Three modern trials guide contemporary practice. The Catheter-Directed Venous Thrombolysis in Acute Iliofemoral Vein Thrombosis (CaVenT) trial was published in 2012 and the Acute Venous Thrombosis: Thrombus Removal With Adjunctive Catheter-Directed Thrombolysis (ATTRACT) trial was published in 2017 [78,79]. In CaVenT, CDT reduced the rate of PTS at 2 years from 55.6% to 41.1% (NNT was 7 (4–502) in 209 patients with acute iliofemoral DVT). Importantly, the effect became more pronounced at 5 years, with 71% of the control group and 43% of the CDT group developing PTS (the NNT dropped to 4 (2–7)) [80]. Severe PTS was present in 1% of the control group and 5% of the CDT group, and quality of life scores did not differ between groups. Procedural bleeding complications occurred in 20 patients (9.6% risk) without any intracranial hemorrhage. The ATTRACT trial randomized 692 patients with acute femoro-popliteal or iliofemoral DVT to PCDT vs. anticoagulation alone, and did not show a significant difference in the rates of PTS at 2 years (47% vs. 48% rates of PTS in the PCDT and control groups). There were significantly more bleeding complications in the intervention group (1.7% vs. 0.3% in the intervention and control groups) but no intracranial hemorrhages occurred. A subgroup analysis of patients with iliofemoral DVTs also failed to show any significant reduction in the risk of PTS or improvement in quality of life measures with PCDT [81]. An analysis of secondary outcomes did show that the proportion of patients with moderate–severe PTS was reduced with PCDT compared to standard treatment (18% vs. 24% (*p* = 0.04)), and this effect was more pronounced in the iliofemoral subgroup (18% vs. 28% (*p* = 0.02)). In the iliofemoral subset, pain and swelling scores at 10 days were significantly lower in the PCDT group (pain score decrease of 1.76 vs. 0.51 (*p* = 0.009) and leg circumference (cm) change of −0.79 vs. 0.22 (*p* = 0.002) in the PCDT and control groups). Echoing the above findings, QOL scores were improved during the first 6 months (but not later) in patients assigned to the PCDT group [82]. Along the same lines, the recent CAtheter Versus Anticoagulation Alone for Acute Primary (Ilio) Femoral DVT (DUTCH CAVA) trial randomized 184 patients with iliofemoral DVT to either PCDT plus standard treatment or standard medical treatment alone. The risk of PTS at one year, defined as two consecutive Villalta scores ≥ 5, was not significantly different between groups (29% vs. 35% in the PCDT and standard treatment groups, respectively, *p* = 0.42). Major bleeding occurred in 5% of the PCDT group and in 0% of the standard treatment group, without any intracranial hemorrhage. The DUTCH CAVA trial did not specifically examine the effect of PCDT on acute leg symptoms, but did find that QOL measures were not significantly different between groups at one year.

Taken together, the CaVenT, ATTRACT and DUTCH CAVA data suggest that locoregional techniques reduce acute DVT symptoms and may also improve early post-DVT QOL. Mid-term (2 year) results are disappointing, but based on CaVenT the benefit of CDT might be more apparent in the very long term with a reduction of the risk of PTS but not of severe PTS. Furthermore, no improvement in quality of life was shown at five years. The use of CDT and PCDT should be restricted to patients with very symptomatic iliofemoral DVT (e.g., massively swollen, to the point of limiting ambulation) and is likely to be beneficial only for acute symptoms and in the very long-term. Other indications for early thrombus removal include phlegmasia cerulea dolens, but in this situation the objective is limb salvage as opposed to prevention of PTS.

### 2.6. Should Early Mobilization Be Recommended to Reduce the Risk of PTS?

Early mobilization may be beneficial for reducing the recurrence and severity of PTS. A small randomized trial with three groups randomized patients to bed rest without compression, early mobilization with ECS or early mobilization with inelastic compression [83]. There was a significant reduction in the mean severity of PTS at 2 years (Villalta scores of 5.1 and 8.2 in the bed rest and early mobilization groups, *p* < 0.01). However, this trial had two independent variables (bed rest and compression) that were both altered. It is impossible to separately analyze the effect of compression compared to that of early mobilization. Given the low risk of harm, we recommend early mobilization to our patients.

The patient in our vignette is at high risk for PTS and we would prescribe him:

- A lead-in course of 15–21 days of LMWH followed by indefinite therapy with a DOAC (unprovoked VTE in a male [12]). There is a theoretical benefit of LMWH over oral anticoagulants, and, given the similar risk profile, we prescribe a short course of LMWH for patients with extensive proximal DVT.
- Below-knee ECS (ideally, 30–40 mmHg, but 20–30mmHg is acceptable to favour compliance) are prescribed as early as possible and continued at least until the patient is no longer symptomatic from a venous symptoms standpoint. As this patient already has signs of chronic venous insufficiency (hyperpigmentation), he should theoretically be prescribed long-term ECS to prevent any worsening of his CVI/PTS.
- CDT or PCDT can be considered if the patient is very symptomatic (massive swelling, can not walk) or remains very symptomatic after several days of anticoagulant and ECS treatment. Locoregional techniques can be beneficial up to 14 days after the DVT, and, therefore, anticoagulation alone with watchful waiting and close follow-up is an acceptable strategy.
- A statin might be considered in the future, but there is not enough evidence to support it currently.
- Early mobilization is recommended and bed rest should be avoided unless there is another contraindication.

## 3. Case Two—Treatment of Post Thrombotic Syndrome

A 73 year old woman with obesity (body mass index: 35 kg/m^2^) and a history of right-sided femoral DVT diagnosed 8 months ago presents with moderate right leg heaviness, cramping, pruritus and pain. These symptoms are worse at the end of the day and relieved by elevation of the leg. On exam, she has moderate hyperpigmentation, erythema, edema and venous ectasia of the right leg. She also has mild edema and venous ectasia on the left. She has been adherent to oral anticoagulation since diagnosis. She is retired and spends the majority of her day watching television.

### 3.1. How Is PTS Diagnosed?

**Figure 1:**
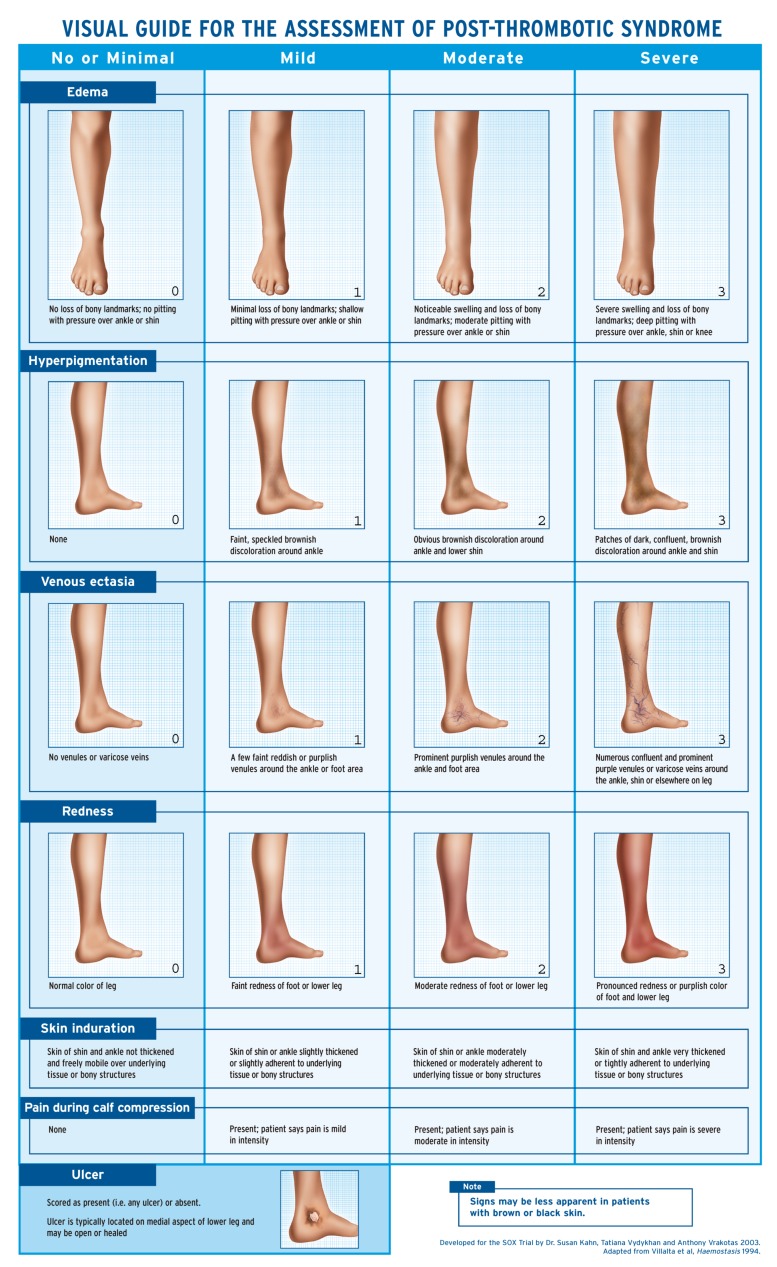
Signs of PTS included in the Villalta scale. Each one is scored out of 3 (with 0 being absent and 3 being most severe). Symptoms are also scored out of 3, and include pain, cramps, heaviness, paresthesia and pruritus. Scores 5–9 represent mild PTS, 10–14 is moderate and ≥ 15 is severe. The presence of a venous ulcer automatically classifies the PTS as severe. The Villalta scale score is non-specific and does not distinguish pre-existing chronic venous insufficiency from PTS.

PTS is a clinical diagnosis based on symptoms and signs. The diagnosis should be delayed by 3–6 months from the index DVT to allow acute DVT symptoms to subside. Three scales have been developed specifically for PTS (Villalta, Ginsberg and Brandjes). The Villalta scale is endorsed by the International Society of Thrombosis and Hemostasis (ISTH) and the American Heart Association (AHA) for the purpose of diagnosing PTS, and is currently widely used [15,84] (Figure 1). Each scale has its own advantages and limitations. The Villalta scale tends to be more sensitive but less specific than the Ginsberg measure [10]. This is because the Villalta scale awards points for a greater number of less-specific symptoms and signs, whereas the Ginsberg measure requires both pain and swelling as well as variation in these symptoms based on activity. The Brandjes scale has only been used in two trials, and is not widely employed. For a detailed review of available rating scales, see Wik et al. [85]. Whichever scale is used, the clinician should keep in mind that none are specific for PTS. For example, it has been shown in the REVERSE study that up to 40% of PTS assessed with the Villalta scale could at least in part be attributable to pre-existing CVI [86].

The patient in our vignette scores at least 15 points on the Villalta scale for her right leg. The score for the left leg is 5 (for heaviness, edema and venous ectasia). She has significant CVI in her left leg but not PTS per se, as she has no history of DVT in this leg. On her right leg she has severe PTS, and her PTS is likely to have been exacerbated by pre-exiting CVI and obesity [87].

### 3.2. Should Lifestyle Interventions Be Prescribed for Patients with PTS?

Lifestyle interventions that are recommended for PTS include physical exercise, weight loss, elevation of the limb at rest and moisturizing creams. Physical exercise that strengthens leg musculature could relieve PTS by improving calf muscle pump function [88]. A randomized trial of 43 patients with PTS showed that a structured 6 month physical exercise program improved venous disease-specific QOL scores (VEINES-QOL improvement an average of 6 points and 1.4 points in the exercise and control groups, respectively, with mean difference 4.6 points, *p* = 0.03) [89]. However, Villalta scores were not significantly different between groups. Obesity is a well-known risk factor for PTS, but there are no studies addressing whether treatment of obesity improves PTS [90]. Weight loss is recommended on the theoretical basis that reduced intra-abdominal pressure and venous compression would lead to reduced venous hypertension in the lower extremities. Elevation of limbs at rest aids with venous drainage by reducing hydrostatic pressure. Studies have shown enhanced microcirculatory flow [91] and reduced venous pooling [92] with limb elevation, but no studies have specifically investigated the role of limb elevation in the setting of PTS. Moisturizing creams can prevent dryness and skin breakdown, which can lead to venous ulcers. We generally recommend the above lifestyle interventions to all our patients, as they have the potential for benefit and a very low risk of harm.

### 3.3. What Medical Measures Should Be Used for Treatment of PTS?

Venoactive medications are commonly used around the world to treat chronic venous symptoms [93,94], but this use is only supported by moderate-quality evidence [95]. RCTs have shown improvement in leg edema as well as symptoms such as cramps, restless legs and paresthesia. However, data supporting the use of venoactive medications in PTS is limited. Venoactive drugs’ mechanism of action is not entirely clear [96]. Rutosides are thought to reduce capillary permeability [97,98]; defibrotides downregulate plasminogen activator inhibitor-1 and upregulate prostaglandin, prostacyclin and thrombomodulin [99]; and flavonoids have a multi-faceted mechanism of action [100]. The micronized purified flavonoid fraction (MPFF) contains a combination of several flavonoids, and is thought to increase venous tone, reduce stasis, reduce venous wall inflammation and increase capillary permeability [101]. As flavonoids target the pathophysiological mechanisms of PTS, they are a particularly interesting candidate for further research. To date, only four randomized trials have investigated venoactive medications for PTS [97,98,99,100]. The limitations of these trials include small sample size (ranging from 29 to 288), short treatment period (ranging from 8 weeks to 12 months), lack of a washout period (in the single crossover trial), lack of reporting on adverse events (in one trial), questionable clinical utility of the outcome measures (calf and ankle circumference in one trial) and lack of adequate blinding (some deficiency in all four trials). As such, meta-analyses have judged the evidence to be of low quality [102,103]. The most recent Cochrane meta-analysis found no evidence to conclude that rutosides are superior to placebo or ECS [102]. Another meta-analysis concluded that there was only limited evidence in support of venoactive medications for treatment of PTS [103].

We do not currently recommend venoactive medications because of the low quality of data supporting their use. Further research is needed to clarify the utility of these medications. Micronized Purified Flavonoid Fraction for the Treatment of Post-Thrombotic Syndrome (MUFFIN-PTS) trial (ClinicalTrials.gov identifier NCT03833024) is a randomized, placebo-controlled trial that will compare micronized purified flavonoid extracts to placebo for the treatment of PTS. We anticipate that it will provide high-quality data regarding the effectiveness and safety of one class of venoactive medication.

### 3.4. Should Compression Therapies Be Used for Patients with PTS?

#### 3.4.1. ECS

Compression stockings are the mainstay of treatment for established PTS [15]. While controversy surrounds ECS for prevention of PTS, clinicians generally accept the role of ECS in the treatment of PTS.

However, the data to support this are limited. Only two trials have evaluated ECS for the treatment of PTS. Ginsberg et al. randomized patients with symptomatic PTS to ECS or placebo stockings [54]. Eleven out of 18 patients (61.1%) in the ECS group and 10/17 patients (58.8%) in the placebo group were considered to have treatment failure (lack of improvement or worsening of leg pain and swelling after three months of treatment); this difference was not significant. Lattimer et al. randomized 34 patients to one of four ECS types (18–21 mmHg vs. 23–32 mmHg, and below-knee vs. above-knee) [104]. All ECS types significantly improved hemodynamic parameters such as the venous filling index, venous volume and time to fill. However, no clinical end points were evaluated. Because of the small sample sizes and contradictory results between studies, a recent Cochrane review judged the evidence to be of very low certainty [105].

Evidence to support the use of ECS is also extrapolated from the CVI literature. Randomized trials comparing ECS to no intervention [106,107], low to high pressure ECS [108,109] and ECS to sclerotherapy [110] have demonstrated relief of symptoms with ECS. Guidelines also recommend ECS for CVI [111]. It is compression in general, and not necessarily ECS, that aids the healing of venous ulcers and prevents further worsening of CVI [112]. Given plausible mechanisms, it is reasonable to assume that ECS would also be beneficial for PTS. In our routine clinical practice, we prescribe ECS for all patients with symptomatic PTS, with a usual initial prescription of 20–30 mmHg (lower than the strength usually prescribed in RCTs for PTS prevention) to favor compliance. This can be escalated to 30–40 mmHg or even higher strengths if the symptoms persist despite good adherence. If there is no improvement at 1–3 months, an alternative diagnosis for the patient’s symptoms should be considered (e.g., peripheral arterial disease, arthritis, neuropathic pain).

#### 3.4.2. Venous-Return Assist Devices

Venous-return assist devices such as the Venowave can be tried for patients with moderate to severe PTS symptoms that are unresponsive to ECS. The evidence behind this is of low certainty, as only two small placebo-controlled, cross-over trials have been conducted [105]. O’Donnell et al. enrolled 32 patients and did not find a difference in the primary end point (composite of reported benefit from the device, at least moderate improvement in symptoms and willingness to continue using the device) [113]. However, they found an improvement in the secondary end points of Villalta scale score (12.2 vs. 15 for the device and placebo treatments, *p* = 0.004) and QOL (venous disease-specific quality of life scores of 52.5 vs. 50.2 for the device and placebo treatments, *p* = 0.004). Ginsberg et al. enrolled 15 patients with intractable PTS and evaluated an extremity pump that applied 50 mmHg of pressure. They found significant improvement of a custom symptom score (*p* = 0.007) [114]. Importantly, 80% of patients (12/15) were considered to have successful treatment.

Overall, the data in support of venous-return assist devices is of low certainty, and the demonstrated benefit has been modest. However, adverse events are uncommon, and patients with moderate to severe PTS with refractory symptoms or those who are unable to wear ECS can be offered a trial of a venous-return assist device.

### 3.5. When Should an Interventional Approach Be Pursued?

Endovenous and open surgical treatment can be considered for a highly selected group of patients with severe PTS [15]. However, high quality data from RCTs to support their use is not available. Possible interventions include stenting for chronic iliac vein obstruction, greater saphenous vein (GSV) ablation for GSV reflux, valve reconstruction for reflux and hybrid procedures [115,116]. Patients with PTS symptoms refractory to conventional management can undergo repeat Doppler ultrasound studies to assess for chronic iliac obstruction and severe GSV reflux.

#### 3.5.1. Venous Deobstruction:

Patients with chronic iliac vein obstruction may benefit from endovascular stenting. A recent meta-analysis of retrospective studies included 1118 patients with chronic iliofemoral venous obstruction who underwent stenting [117]. Razavi et al. found a 79% one year primary patency rate as well as complete pain relief in 69%, complete edema relief in 64% and complete ulcer healing in 71%. Complication rates were 0.9% for major bleeding, 0.6% for pulmonary embolism and 0.3% for periprocedural mortality. Several RCTs will be starting soon to assess the benefit of venous deobstruction in patients with proximal occlusion causing moderate to severe PTS.

#### 3.5.2. Venous Reflux

Patients with greater saphenous vein reflux may benefit from endovascular ablation, surgical stripping or sclerotherapy [118,119]. This may improve PTS symptoms by redirecting blood through a competent deep venous system. If there is concomitant iliac vein obstruction, it should be addressed first. It is important to note that VTE often occurs in patients with pre-existing venous reflux. Superficial reflux is about 5 times more common in patients with VTE than in controls [120]. Patients with refractory symptoms from PTS can be investigated with Doppler ultrasound to assess for co-existing reflux, which may be amenable to treatment. Lastly, patients with reflux can sometimes benefit from surgical valve reconstruction.

It should be emphasized that none of the above interventional techniques are supported by high-quality data, and that patients under consideration for these should be referred to a center with extensive expertise in the field. Patients should be carefully selected for these procedures. 

The patient in our vignette has severe PTS with a Villalta score > 15. She would be treated as follows:

- Leg elevation on a stool while sitting, weight loss with an ideal target BMI < 25 kg/m^2^, initiation of a structured exercise program to strengthen calf muscles, and skin moisturizers.
- ECS 20–30 mmHg with progression to 30–40 mmHg or even higher if not effective, and potential progression to a venous-return assist device if still not effective.
- Repeat ultrasound imaging can be considered to assess for iliac vein obstruction and greater saphenous vein reflux. If either is found, referral can be made to a center with expertise in iliac stenting or greater saphenous vein ablation.
- No venoactive medications.

## 4. Conclusions

Several prediction models exist for PTS, and these provide a fair estimate of a given patient’s risk of developing PTS after DVT. They are especially useful for estimating whether a patient falls on the high- or low-risk side. High-risk patients can then be followed closely and their modifiable risk factors should be optimized.

In terms of PTS prevention, anticoagulation with good medication adherence is the most important tool (Table 2). If a VKA is chosen, careful INR monitoring is critical. LMWH have anti-inflammatory properties, and may be superior to VKA, whereas the effectiveness of DOACs in this setting is uncertain. The efficacy of ECS for prevention of PTS remains equivocal and ECS should be prescribed for acute symptoms, CVI, and depending on clinician and patient preference. Locoregional interventional therapies likely reduce acute DVT symptoms and long-term (5 year) PTS risk, but only for high-risk patients (e.g., those with extensive proximal DVT). After PTS is established, ECS are the mainstay of treatment. Venoactive medications and interventional approaches require additional evaluation before they can be recommended.

Future directions for research include determining whether anti-inflammatory properties of LMWH or of statins translate to a reduced incidence of PTS, establishing whether ECS prevents PTS in a placebo-controlled trial with high adherence, and determining whether venoactive medications are useful for treating PTS.

## Figures and Tables

**Table 1:** Post-thrombotic syndrome (PTS) risk prediction models. BMI—body mass index; NSAID—non-steroidal anti-inflammatory drug; DVT—deep vein thrombosis. Rabinovich model: 0 points—6.4%, 1 point—13.4%, 2 points—16.4%, 3 points—25%, ≥4 points—30% risk of PTS; Amin model: 0–2 points—10%, 3–4 points—20%, ≥5 points—40% risk of PTS; Méan model—0–3 points—24.4%, 4–5 points—38.4%, ≥6 points—80.7%. In the Méan model, one point is awarded for each of the following leg symptoms and signs: pain, cramps, heaviness, pruritus, paresthesia, edema, skin induration, hyperpigmentation, venous ectasia, erythema, pain during calf compression.

Rabinovich Model		Amin Model		Méan Model	
Category	Points	Category	Points	Category	Points
BMI > 35	2	Age > 56	2	Age ≥ 75	1
Iliac vein thrombosis	1	BMI > 30	2	Prior varicose vein surgery	1
Villalta scale score in moderate/severe range at baseline	1/2	Varicose veins	4	Multi-level thrombus	1
		Iliofemoral DVT	1	Number of leg symptoms and signs (up to 11)	1 per symptom/sign
		Provoked DVT	1	Concomitant NSAID/antiplatelet	1
		History of DVT	1		
		Smoking	1		
		Female gender	1		
Patient in Case 1					
Probability of PTS	Points	Probability of PTS	Points	Probability of PTS	Points
25%	3	40%	6	80.7%	6

**Table 2:** Summary of recommendations.

**Prevention of PTS**
1. Anticoagulation should be used for prevention of PTS (strong recommendation, moderate-quality evidence)
2. ECS can be considered for prevention of PTS (weak recommendation, low-quality evidence)
3. Locoregional techniques can be considered in patients with extensive proximal VTE and high symptom burden (e.g., unable to weight bear) for prevention of PTS up to 2 weeks after the acute event (weak recommendation, low-quality evidence)
**Treatment of established PTS**
1. ECS should be used for treatment of PTS (strong recommendation, moderate-quality evidence)
2. Weight loss, calf strengthening, limb elevation at rest and early mobilization can be used for treatment of PTS (weak recommendation, low-quality evidence)
3. Venous return assist devices can be considered for PTS refractory to ECS (weak recommendation, low-quality evidence)
4. Interventions such as de-obstruction, GSV stripping and surgical repair can be considered for treatment of refractory PTS in patients with chronic iliac vein obstruction or GSV reflux (weak recommendation, low-quality evidence)
